# Evaluating the diversity, distribution patterns and habitat preferences of *Carex* species (Cyperaceae) in western Canada using geospatial analysis

**DOI:** 10.3897/BDJ.13.e144840

**Published:** 2025-04-30

**Authors:** Vladimir V. Kricsfalusy, Austin Godfrey, Kakon Chakma, Andrew Stewart, Ivan M. Danylyk

**Affiliations:** 1 University of Saskatchewan, Saskatoon, Canada University of Saskatchewan Saskatoon Canada; 2 Flora of Saskatchewan Association, Regina, Canada Flora of Saskatchewan Association Regina Canada; 3 National Academy of Sciences of Ukraine, Lviv, Ukraine National Academy of Sciences of Ukraine Lviv Ukraine

**Keywords:** sedges, biogeography, species diversity, species hotspots, environmental requirements, Canada

## Abstract

Sedge (*Carex*) is a highly diversified genus of vascular plants with high species diversity in cold-temperate areas of the Northern Hemisphere. In Canada, 313 species of *Carex* are documented with 105 species in Saskatchewan, making it the largest genus of vascular plants in this Province. Research on the distribution and ecology of sedges in Saskatchewan is extremely limited. This study aims to find the distribution patterns of *Carex* species and identify their habitat preferences relative to environmental conditions in Saskatchewan through the application of GIS spatial analysis tools. Data on specimen-based occurrences of *Carex* species were collected, validated and consolidated from the Flora of Saskatchewan Association (FOSA) and analysed along with *Carex* datasets mobilised by the Global Biodiversity Information Facility (GBIF), resulting in 2655 individual records of occurrences. Our research includes seven environmental variables to explore relationships between *Carex* species and environment. The study produced comprehensive spatial maps and graphs illustrating species occurrences, species richness and diversity hotspots. It was found that *Carex* species have a diverse habitat preference strongly associated with temperature and precipitation and, to a lesser extent, soils. The species occurrences are mostly concentrated in the Boreal Plain and Prairie ecozones of the Province. Notably, species richness peaked in the central part of Saskatchewan in areas with moderate elevation and temperature and high precipitation. This integrative analysis emphasises the need for region-specific assessments to effectively manage and preserve biodiversity.

## Introduction

Effective mitigation of biodiversity loss requires approaches that integrate large-scale geographic distribution, digitised specimen data and complex spatial analysis. The application of Geographic Information Systems (GIS) and Geoinformatics in biodiversity assessment at local and regional scales has been highlighted in several studies (Murthy et al. 2003, Salem 2003, Kricsfalusy et al. 2015, Asaad et al. 2016, Soltis and Soltis 2016). These studies offer valuable insight into data sourcing and exploration for biodiversity analysis and big data management, as well as GIS and geoinformatics procedures for delineating conservation gaps. Mapping the species distribution using GIS could provide better understanding of their diversity and richness that are key components in conservation planning. Several publications have shown (see review by Franklin and Miller (2010)) that data-driven assessments give important spatial-temporal information, offering comprehensive perspectives on studying and monitoring biodiversity across various scales, as well as forecasting the impacts of global environmental changes at species and ecosystem level.

Contemporary species ranges were formed during the long-term process of evolution under the influence of the complex action of environmental factors and climatic changes (Sexton et al. 2009). The general patterns of distribution of species will be expressed to a greater extent by the size of their ranges, from which it is also possible to delineate the centres of species diversity and, in part, their regional specificity. By evaluating the general limits of species ranges, it is possible to assume with a high degree of reliability how significant the diversity of species will be in one or another region.

Sedge (*Carex* L., Cyperaceae) is a highly diversified genus of vascular plants, comprising over 2000 species worldwide, predominantly found from the temperate to arctic zones (Ball and Reznicek 2002). According to recent studies (Escudero et al. 2012, Martin-Bravo et al. 2019), this genus has a nearly cosmopolitan distribution, displaying an inverted latitudinal richness gradient with high species diversity in cold-temperate areas of the Northern Hemisphere. There are 313 species of *Carex* in Canada (Brouillet et al. 2010) and 175 species in the Prairie Provinces (Alberta, Saskatchewan and Manitoba), with 105 species in Saskatchewan, making it the largest genus of vascular plants in this Province (Leighton 2012). Considering the broad distribution of the genus *Carex* and the fairly large size of its species' ranges, sedges can serve as a model group for studying not only biogeographic, but also ecological determinants that shape plant diversity.

Despite the wide distribution, sedges are not immune to threats, with habitat loss posing a significant challenge to their survival. The loss of *Carex* habitats raises concerns about biodiversity and the stability of ecosystems they support. It was shown that the crucial step in setting conservation priorities requires identifying and evaluating biodiversity hotspots (Yu et al. 2017). Traditionally, biodiversity hotspots are identified using species richness, which highlights areas with the highest number of species, simplifying conservation planning with limited information and resources (Qian et al. 2022).

Sedge-rich communities are important wetland habitats and have been a focal point of restoration research in temperate North America and the Arctic (Kettenring et al. 2006). Despite the prominent role of *Carex* species in ecosystems, sedge ecology is poorly studied (Gignac et al. 2004, Barrett 2013, Waterway et al. 2016) and no review of sedge ecology in Saskatchewan exists. Our previous work (Danylyk and Kricsfalusy 2020) analysed the phytogeographical differentiation of the genus *Carex* in this Province applying the principles of botanical and geographical zoning of the world.

This current research aims to identify the distribution patterns of *Carex* species, delineate their diversity hotspots and determine the bioclimatic conditions that influence species richness and their habitat preferences in Saskatchewan through the application of GIS spatial analysis tools. This may offer essential information needed to implement better conservation strategies for this taxonomic group as well as to forecast the impacts of global environmental changes on species and ecosystem level.

## Material and methods

### Study area

**Figure 1.**
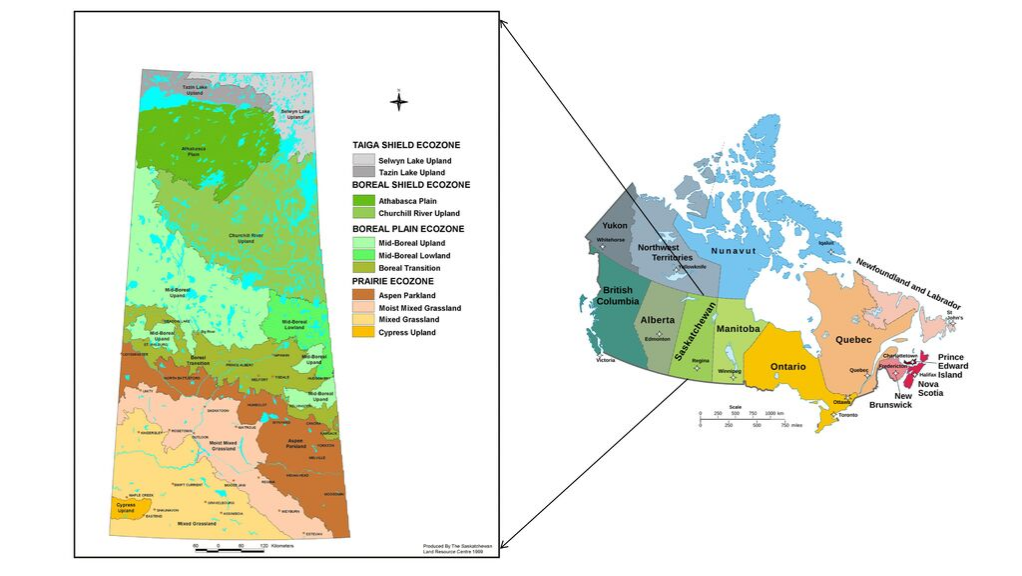
Location of Saskatchewan depicting four ecozones or biomes in the Province with reference to Canada (Source: https://www.friendlyforest.ca/Page_files/Sask_Eco_Regions_Map.htm).

Saskatchewan is one of the Prairie Provinces of western Canada (Fig. 1) with a total area of 651,036 km^2^ of which 591,670 km^2^ is land, while 59,366 km^2^ is water (Anonymous 2008). GPS coordinates of the Province are as follows: 52.9399° N, 106.4509° W. From north to south, there are four ecological zones or biomes (Taiga Shield, Boreal Shield, Boreal Plain and Prairie) which are further subdivided into 11 ecoregions (Acton et al. 1998). The Taiga Shield ecozone stretches across part of Canada’s subarctic. This ecozone is dominated by the boreal coniferous forest. The Boreal Shield ecozone is widely forested with black spruce forming mixed stands with jack pine and tamarack. This ecozone separates the warmer Boreal Plain to the south from the colder Taiga Shield to the north. The Boreal Plain ecozone is characterised by mixwood and coniferous forest. The deciduous component is represented along the southern boundary adjoining the prairie grasslands. The Prairie ecozone is part of the Interior Plains of Canada, which are a northern extension of the Great Plains of North America.

Saskatchewan has a continental climate, experiencing extremes in temperature and weather events, relatively low precipitation which falls mostly during summer months and considerable sunshine. Temperatures can range from -40°C in the winter to +35°C during summer. There is a significant difference in mean temperature and precipitation between the southwest and northeast parts of the Province. Generally, mean annual temperatures drop from south to north and from west to east within the Province. Conversely, precipitation levels ordinarily increase from south to north. Overall, there are warmer, drier conditions in the southwest and cooler moister conditions in the northeast of Saskatchewan.

### Data validation

**Figure 2.**
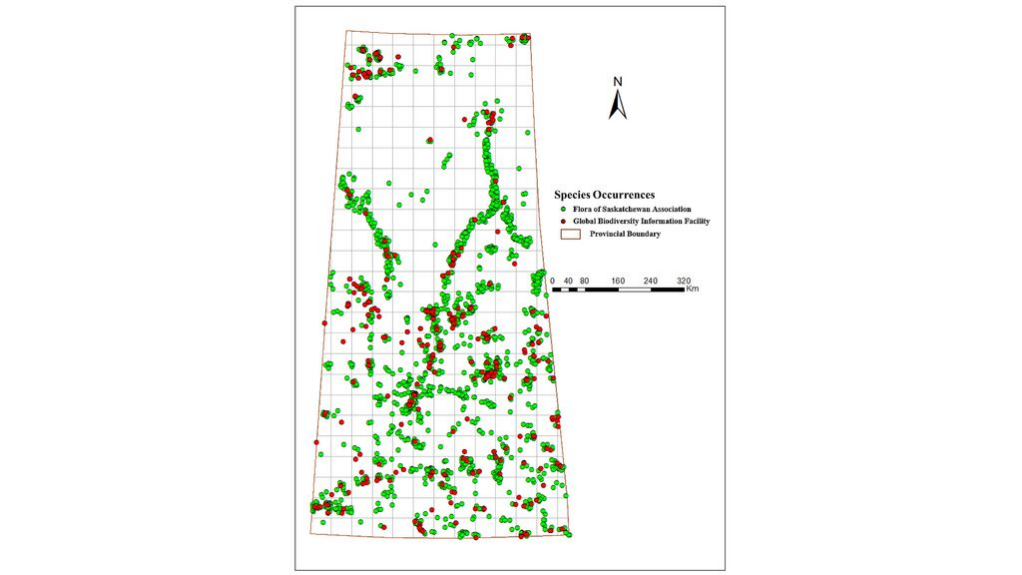
Occurrences of *Carex* species in Saskatchewan representing 2084 specimens in the Flora of Saskatchewan Association (FOSA) dataset and 571 specimens in the Global Biodiversity Information Facility (GBIF) datasets.

The process of validation entailed taxonomic verification of *Carex* species against the Plants of the World Online (P.O.W.O. 2024) and the Database of Vascular Plants of Canada or VASCAN (Brouillet et al. 2010). The data filtering of the *Carex* specimen-based occurrences was conducted to eliminate multiple entries by cross-checking the Flora of Saskatchewan Association (FOSA 2022) dataset that contains 2207 records with the Global Biodiversity Information Facility (GBIF 2020) datasets that includes 940 records. To deal with the bias and mistakes in the occurrence data, several processes to manipulate the raw data were conducted. First, records that had either no geographical coordinates or unclear taxonomic information were excluded. Second, records that have duplicated coordinates were removed. Each validated species includes georeferenced points with an attribute table consisting of the sampling locations linked to the FOSA and GBIF datasets. In total, 2655 records of 105 species were validated for this study, 2084 records from FOSA and 571 records from GBIF (Fig. 2). It should be noted that all produced maps represent the record count (herbarium vouchers) and, as such, are a function of collecting bias. These maps may not be necessarily representative of the true *Carex* species distribution in the Province.

Moreover, the study integrated expert ecological knowledge into the validation process. Patterns observed in the interpolated maps were cross-referenced with published literature on *Carex* species in Saskatchewan. This approach allowed for qualitative validation of the spatial patterns, ensuring that the interpolated richness hotspots aligned with ecologically plausible areas given the environmental gradients across the Province. The integration of expert-driven validation adds a unique layer of confidence to the results, as it combines quantitative spatial analysis with ecological expertise to minimise misinterpretation of the interpolated data.

### Environmental variables

Environmental datasets are the independent variables of GIS layers developed for Saskatchewan (Kricsfalusy et al. 2015). These include raster and feature layers of topography (elevation), climate (precipitation, temperature and climate moisture index), soil type and ecological systems (ecozone and ecoregion). These variables (Table 1) were converted into point features, classified into ranges and spatially joined with the *Carex* occurrence point features such that each of the species’ occurrences carries the generated values for each of the environmental parameters. Thus, seven derivatives of validated species’ layers with environmental parameter features’ columns form the parameter for analysis. Each parameter was broken down into specific categories which were analysed using box plot of species occurrences, bar chart of total species count, bar chart of grids, species count map and hotspots map.

### Data analysis

Applying GIS spatial and statistical tools, the patterns of the *Carex* specimen-based occurrences were assessed. A hotspot analysis that emphasises areas of species richness was completed, based on unique species counts per each grid and the number of grid cells occupied by each species. Saskatchewan was divided into 325 UTM grid cells of 50 km x 50 km (Kricsfalusy et al. 2015, Kricsfalusy 2021), over which the studied species were rendered for analysis. For the hotspot rendition, species richness (the unique species counts) of a grid cell provides information for the diversity indices of the cell. Species richness (*r*), frequency (*Fr*) and the Shannon-Weiner diversity index (*H*) were calculated (Begon et al. 1986) for each ecozone and ecoregion.

This study applied the ESRI ArcGIS platform to engage the environmental parameters’ raster and the *Carex* species points features in spatial analysis. In this study, the join attribute features refer to the environmental variable values associated with *Carex* occurrence points, while the target features represent the georeferenced *Carex* species occurrences with unique species counts and diversity metrics. The completed GIS analysis procedures began with Spatial Join exercises that connect analysis feature layers, based on their spatial relationship. The Join Attribute table includes the Join_ID column that shows the value of joined features attributes and the Target_ID that constitute the primary values for analysis. Two Spatial Join processes were conducted and during each of the sessions, the Join-One-to-One Spatial Join option and the Closest Match option were selected to ensure that each of the species feature points of occurrences assume the various values of the environmental parameters. Statistical analysis engaged the frequency column to produce the frequency for each unique combination of the specified attribute field before the Inverse Distance Weighting (IDW) of the geostatistical tool was used to produce interpolated surfaces of the unique species distribution pattern as in the case of diversity hotspot analysis.

### Data visualisation

Boxplots were used to compare the *Carex* specimen-based occurrences across categories of environmental variables, while bar graphs illustrated species richness and the number of grids within each variable category. Data visualisation for hotspots analysis is enhanced by the Spatial Join of validated species points derivatives with grid cells and are rendered using a spectrum of colour to indicate the pattern of the species spatial distribution. A more dense colour indicates a higher species count in grid cells which are linked to favourable conditions for *Carex* species. The interpolated surfaces used the colour gradient ranges from blue (low species richness) to red (high species richness), delineating areas with varying levels of diversity.

## Data resources

A list of the *Carex* specimen-based occurrences in Saskatchewan is available from the Global Biodiversity Information Facility (GBIF 2020) and the Flora of Saskatchewan Association (FOSA 2022). The GBIF datasets include 940 records sourced on 23 October 2020 (https://doi.org/ 10.15468/di .gu7gqs). The data from the FOSA (http://floraofsaskatchewan.ca) were sourced on 18 February 2022. The obtained dataset named FosaCarex-Excel contains 2207 records of *Carex* species in Saskatchewan. These occurrence records represent the collection of herbarium specimens at W.P. Fraser Herbarium at the University of Saskatchewan (SASK 2022). To obtain access to the dataset, a formal request should be made to the FOSA board (floraofsask@gmail.com).

## Results

### Spatial distribution of Carex species

**Figure 3.**
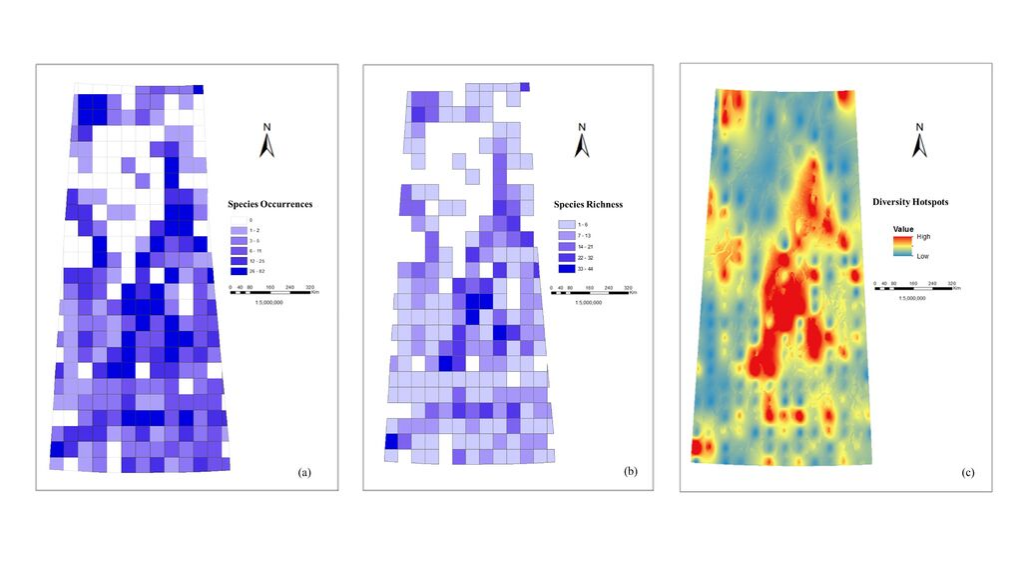
Spatial distribution of the *Carex* specimen-based occurrences in Saskatchewan: **a** species occurrences with maximum of 82 collections within a grid cell; **b** species richness with maximum of 44 species within a grid cell; **c** interpolated species hotspots.

The obtained statistics data are presented in Suppl. material 1. A series of charts was developed to illustrate the distribution of the *Carex* specimen-based occurrences within each of the grid cells (Fig. 3a) with five ranges of occurrences and the unique species counts within the grid cells (Fig. 3b). The study has revealed that there are cells with a species record as high as 82 and species richness (the total number of species) as high as 44. The study has shown that *Carex* species occur in 253 grid cells (77%) of the total 325 grid cells of the Province.

The *Carex* hotspots that indicate the areas of Saskatchewan with the highest species count are delineated not by the frequency of occurrences, but by the species richness within a given grid cell. The analysis of *Carex* species counts yielded five classes which were obtained using Jenks Natural Breaks classification method (Dagher-Kharrat et al. 2018). These classes represent the different levels of species cluster rate in each grid cell (Fig. 3b). The classes range from 1-6 counts (the lowest range that renders species sparseness) to 33-44 counts (the highest range of species hotspots). The blank grid cells indicate where there are no species occurrence records. The hotspot analysis considered the species ranges of 22-32 and 33-44 to provide information for areas with hotspots of *Carex* species that have been classified into three ranges. The interpolated surface (Fig. 3c) highlights areas with varying levels of diversity.

### Environmental relationships of Carex species

**Figure 4.**
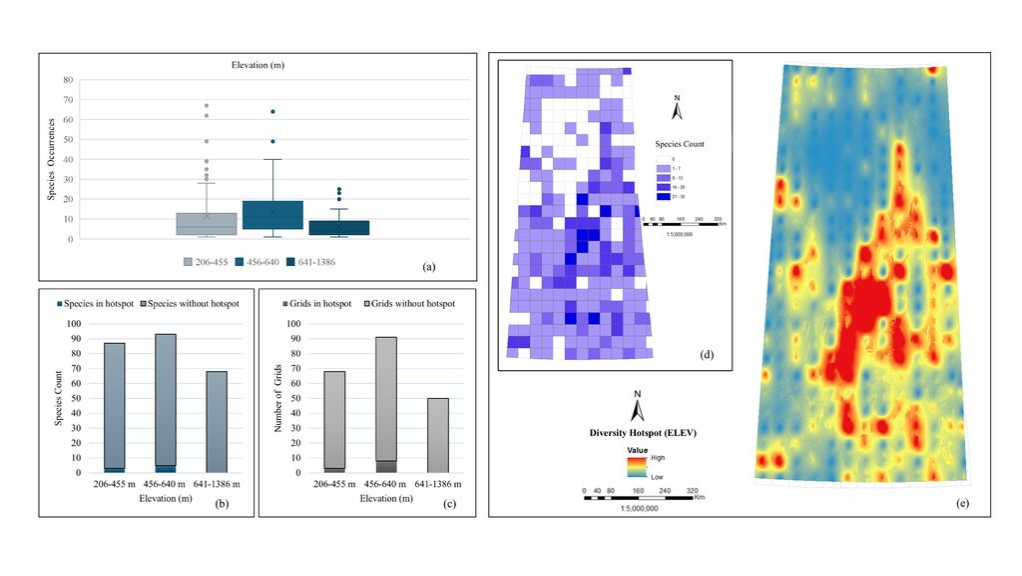
Relationship between *Carex* species distribution and elevation: **a** box plot of occurrences; **b** bar chart of total species count including hotspots and without hotspots; **c** bar chart of total number of grids including hotspots and without hotspots; **d** unique species count per grid; **e** interpolated species hotspots.

*Elevation (ELEV).* The box plot (Fig. 4a) highlights the variation in the *Carex* specimen-based occurrences across different elevations, with the middle elevation range (456-640 m) showing the highest median occurrence and the widest spread, suggesting it supports a diverse group of species. The low (206-455 m) and high (641-1386 m) elevation ranges exhibit more variability in the species occurrences, with some of them being highly prevalent at lower elevations. In the case of species count (Fig. 4b), the highest number of species (93) is observed in the 456-640 m range, followed by 206-455 m (87 species) and the lowest number in the 641-1386 m range (68 species). The middle elevation range (456-640 m) has five species in hotspots, while the low elevation range (206-455 m) has three species in hotspots and the high elevation range (641-1386 m) has no species in hotspots. Grids evaluation shows (Fig. 4c) their highest number (91 grids) in the middle elevation range (456-640 m) where eight grids are in hotspots. The low elevation range (206-455 m) has 84 grids with three grids in hotspots and none in the high elevation range (641-1386 m). The grid map (Fig. 4d) shows a species count in each grid cell which are linked to favourable elevation for *Carex* species. The interpolated diversity hotspot map (Fig. 4e) shows that the central part of Saskatchewan possesses the highest species number, which likely corresponds to the middle elevation range (456-640 m). Thus, all evaluations collectively suggest that *Carex* species are mostly distributed in the range of 456-640 m.

**Figure 5.**
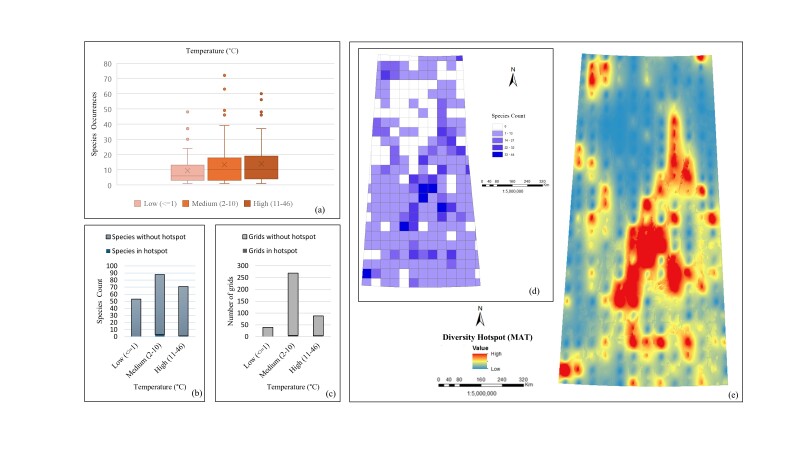
Relationship between *Carex* species distribution and temperature: **a** box plot of occurrences; **b** bar chart of total species count including hotspots and without hotspots; **c** bar chart of total number of grids including hotspots and without hotspots; **d**) unique species count per grid; **e** interpolated species hotspots.

*Temperature (MAT)*. The box plot (Fig. 5a) shows the variability in *Carex* species occurrences across different temperature ranges, with high temperatures (11-46°C) showing the widest species spread (1 to 60), suggesting this range supports a diverse group of sedges. For the medium temperature range (2-10°C), species occurrences vary from 1 to 72. For some species being highly prevalent under colder conditions as for low temperatures (<=1°C), the minimum occurrence is one, the maximum is 48. In the case of species count (Fig. 5b), the highest species number is observed in the medium temperature range (2-10°C) with 88 species, followed by the high temperature range (11-46°C) with 71 species and the lowest in the low temperature range (<=1°C) with 54 species. The medium temperature range (2-10°C) has three species in hotspots, while the high temperature range (11-46°C) has one species in hotspots and the low temperature range (<=1°C) has no species in hotspots. Grids evaluation (Fig. 5c) shows the highest number of grids present in the medium temperature range (2-10°C) with 269 grids, where six grids are in hotspots. The high temperature range (11-46°C) has 88 grids with four grids in hotspots and the low temperature range (<=1°C) has 40 grids with none in hotspots. The species count map (Fig. 5d) shows the number of species in each grid cell, indicating the highest species number linked to favourable temperatures for *Carex* species. The interpolated diversity hotspot map (Fig. 5e) reveals that the central part of the study area has the highest species number, likely corresponding to the medium temperature range (2-10°C). All evaluations collectively suggest that *Carex* species are mostly distributed in the medium temperature range (2-10°C).

**Figure 6.**
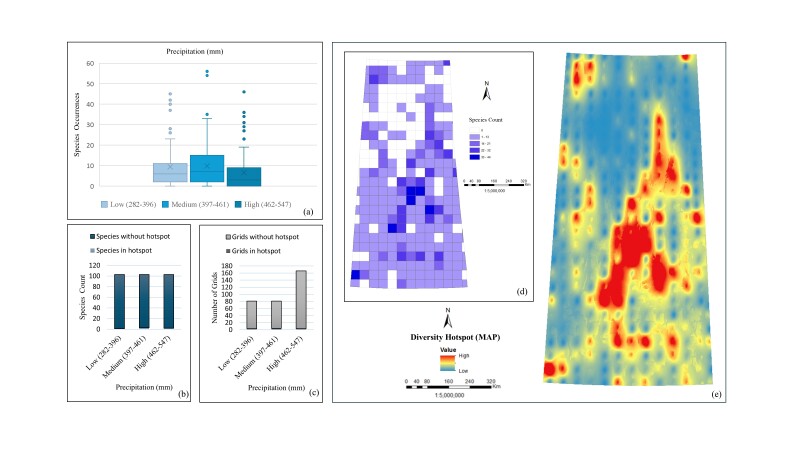
Relationship between *Carex* species distribution and precipitation: **a** box plot of occurrences; **b** bar chart of total species count including hotspots and without hotspots; **c** bar chart of total number of grids including hotspots and without hotspots; **d** unique species count per grid; **e** interpolated species hotspots.

*Precipitation (MAP)*. The box plot (Fig. 6a) presents the variability in *Carex* species occurrences across different precipitation ranges, with medium precipitation (397-461 mm) showing the highest median occurrence and the broadest spread, suggesting this range supports a diverse group of species. Low (282-396 mm) and high (463-547 mm) precipitation ranges exhibit more variability in species occurrences, with some species being highly prevalent under these conditions. In the case of species count (Fig. 6b), the highest species number is observed in the medium precipitation range (397-461 mm) with 93 species, followed by the low precipitation range (282-396 mm) with 89 and the high precipitation range (463-547 mm) with 71 species. The medium precipitation range (397-461 mm) has three species in hotspots, followed by the high precipitation range (462-547 mm) with two species and the low precipitation range (282-396 mm) with one species in hotspots. Grid evaluation (Fig. 6c) shows the highest number of grids present in the high precipitation range (462-547 mm) with 166 grids, where three grids are in hotspots. Both the low (282-396 mm) and medium (397-461 mm) precipitation ranges have 81 grids, with three grids in hotspots for the low range and four grids in hotspots for the medium range. The species count map (Fig. 6d) illustrates their number in each grid cell, indicating the highest species counts linked to favourable precipitation conditions for *Carex* species. The interpolated diversity hotspot map (Fig. 6e) demonstrates that the central part of Saskatchewan, likely corresponding to the high precipitation range (462-547 mm), possesses the highest species number. Therefore, all evaluations collectively suggest that *Carex* species are uniformly distributed across all precipitation ranges, with a slight preference for the high precipitation range in terms of grid density and species number in hotspots.

**Figure 7.**
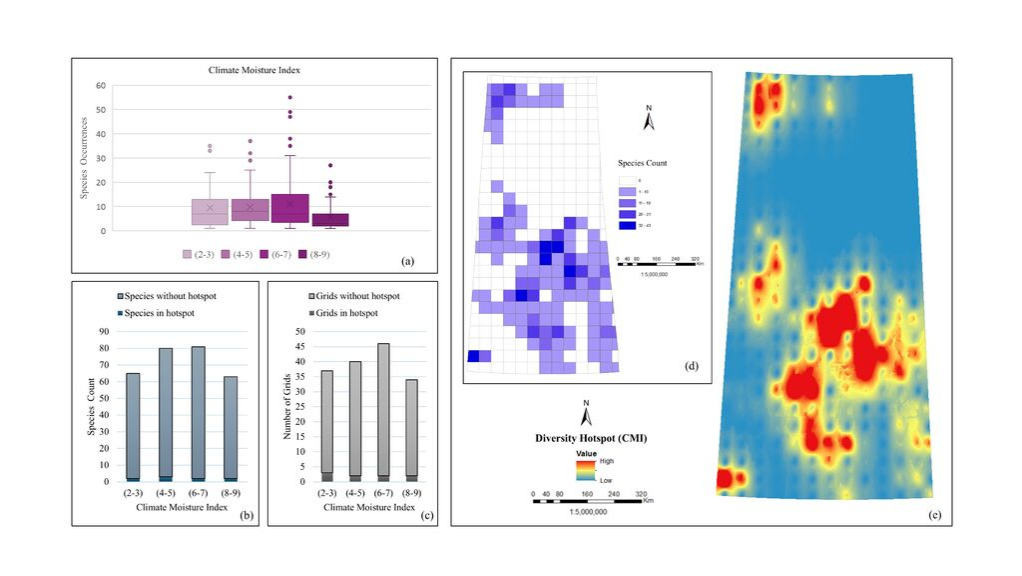
Relationship between *Carex* species distribution and Climate Moisture Index: **a** box plot of occurrences; **b** bar chart of total species count including hotspots and without hotspots; **c** bar chart of total number of grids including hotspots and without hotspots; **d**) unique species count per grid; **e** interpolated species hotspots.

*Climate Moisture Index (CMI).* The box plot (Fig. 7a) underscores the variability in *Carex* species occurrences across different climate moisture index ranges, with the 6-7 category showing the highest median occurrence and the broadest spread, suggesting this range supports a diverse array of species. Lower and higher climate moisture index ranges exhibit more variability in species occurrences, with some species being highly prevalent under these conditions. In the case of species richness (Fig. 7b), the highest species count is observed in the range of 6-7 with 81 species, followed by the range of 4-5 with 80 species, the range of 2-3 with 65 species and the lowest in the range of 8-9 with 63 species. The range of 4-5 has two species in hotspots, followed by the ranges of 2-3, 6-7 and 8-9, each with one species in hotspots. Grid evaluation (Fig. 7c) shows the highest number of grids are present in the range of 6-7 with 46 grids, where two grids are in hotspots. The range of 4-5 has 40 grids with two grids in hotspots, the range of 2-3 has 37 grids with three grids in hotspots and the range of 8-9 has the lowest number of grids, 34, with two grids in hotspots. The species count map (Fig. 7d) illustrates the species number in specific grid cells, indicating higher species counts linked to favourable climatic conditions for the study sedges. The interpolated diversity hotspot map (Fig. 7e) highlights the central part of the Province with the highest species number, likely corresponding to the range of 6-7. Thus, all evaluations collectively suggest that *Carex* species are mostly distributed in the areas with the climate moisture index range of 6-7 and significantly represented in the range of 4-5 as well.

**Figure 8.**
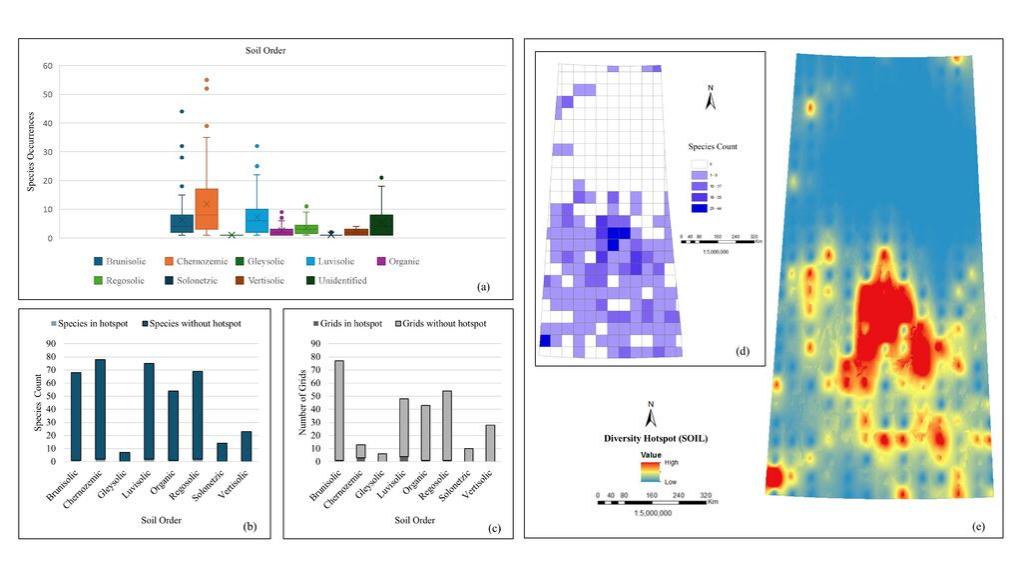
Relationship between *Carex* species distribution and soils: **a** box plot of occurrences; **b** bar chart of total species count including hotspots and without hotspots; **c** bar chart of total number of grids including hotspots and without hotspots; **d** unique species count per grid; **e** interpolated species hotspots.

*Soils (SOIL).* The box plot (Fig. 8a) shows the variability in *Carex* species occurrences across different soil orders, with the Chernozemic soils showing the highest median occurrence and the broadest spread, suggesting this soil type supports a diverse range of species. Other soil orders exhibit varying degrees of species occurrences, with some showing significant variability and others showing more consistent, lower occurrences. Analysis of species distribution (Fig. 8b) shows that their highest count is observed in the Chernozemic order with 78 species, followed by the Luvisolic order with 75 species. The Brunisolic and Regosolic orders also show a significant species number with 68 and 69 species, respectively. The lowest species counts are observed in the Gleysolic (7 species) and Solonetzic (14 species) orders. The Chernozemic and Luvisolic orders each have two species in hotspots, while the Brunisolic and Organic orders each have one species in hotspots. Grid evaluation (Fig. 8c) reveals the highest number of grids present in the Brunisolic order with 77 grids, where one grid is in a hotspot. The Regosolic order follows with 54 grids, including one grid in a hotspot. The Luvisolic order has 48 grids, with four grids in hotspots and the Organic order has 43 grids, with one grid in a hotspot. The Chernozemic order has fewer grids (13), but a higher species count per grid, with three grids in hotspots. The Gleysolic, Solonetzic and Vertisolic orders have the lowest number of grids, with no hotspots. The species count map (Fig. 8d) illustrates their number in each grid cell, indicating the highest species counts linked to favourable soil conditions for the study sedges. The interpolated diversity hotspot map (Fig. 8e) highlights areas of the highest species number in the central part of Saskatchewan, likely corresponding to Chernozemic and Luvisolic soils. All evaluations collectively suggest that *Carex* species are mostly distributed in the Chernozemic and Luvisolic orders, with significant representation in the Brunisolic and Regosolic orders as well.

**Figure 9.**
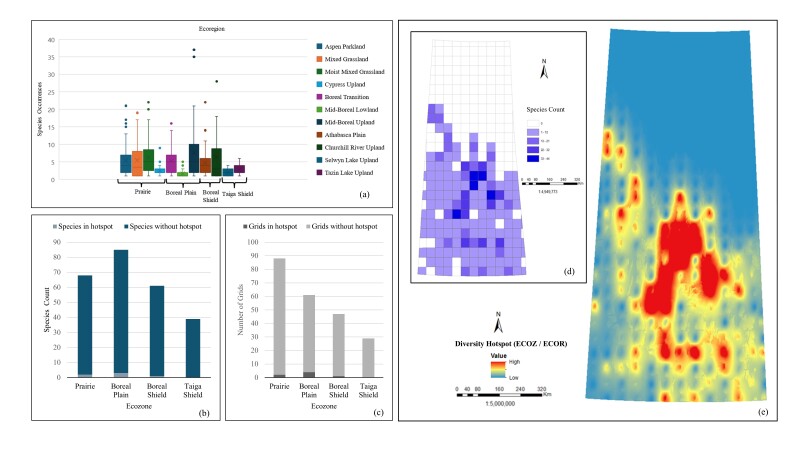
Relationship between *Carex* species distribution and major ecosystems: **a** box plot of occurrences; **b** bar chart of total species count including hotspots and without hotspots; **c** bar chart of total number of grids including hotspots and without hotspots; **d** unique species count per grid; **e** interpolated species hotspots.

*Ecozone and Ecoregion (ECOZ/ECOR).* The box plot (Fig. 9a) underscores the variability in *Carex* species occurrences across different ecoregions, with the Mid-Boreal Upland showing the highest median occurrence and the broadest spread, suggesting this ecoregion supports a diverse range of species. The Churchill River Upland and Aspen Parkland also show significant species occurrences with some variability. Ecoregions, such as the Cypress Upland, Mid-Boreal Lowland, Selwyn Lake Upland and Tazin Lake Upland, exhibit lower species occurrences with less variability. The Boreal Transition (71 species) and Mid-Boreal Upland (70 species) show the highest diversity, indicating these regions offer diverse habitats and favourable ecological conditions. Moderate species counts are observed in the Aspen Parkland (55 species), Moist Mixed Grassland (57 species) and Cypress Upland (42 species), while the lowest counts are in the Athabasca Plain (36 species), Churchill River Upland (28 species), Selwyn Lake Upland (31 species) and Tazin Lake Upland (30 species), likely due to harsher climates or less diverse habitats. The Boreal Plain ecozone has three species in hotspots, the Prairie two, the Boreal Shield one and the Taiga Shield zero (Fig. 9b). Grid evaluation (Fig. 9c) shows the highest number of grids present in the Prairie ecozone with 88 grids, where two grids are in hotspots. The Boreal Plain ecozone follows with 61 grids, including four grids in hotspots. The Boreal Shield ecozone has 47 grids, with one grid in a hotspot and the Taiga Shield ecozone has the lowest number of grids 29, with no grids in hotspots. The species count map (Fig. 9d) indicates the highest species number linked to favourable ecozone conditions for *Carex* species. The interpolated diversity hotspot map (Fig. 9e) identifies the central part of the Province with the higher species count, likely corresponding to the ecoregions within the Boreal Plain ecozone. This map visualises the trend observed on the other charts and maps, indicating that species distribution is more concentrated in certain ecozones. All evaluations collectively suggest that *Carex* species are mostly distributed in the Boreal Plain and Prairie ecozones, with significant representation in the Boreal Shield ecozone as well.

### Habitat preferences of Carex species

The data on the distribution of the *Carex* specimen-based occurrences across multiple ecoregions within four distinct ecozones or biomes (Prairie, Boreal Plain, Boreal Shield and Taiga Shield) are summarised in Suppl. material 2. Each ecozone is further divided into specific ecoregions (totalling to 11), providing a detailed picture of *Carex* species distribution in Saskatchewan.

In the Prairie ecozone, a high number of *Carex* species is notable, with the Aspen Parkland having 55 species, Moist Mixed Grassland 57, Mixed Grassland 39 and Cypress Upland 42 species. Twenty-five species were noted as present within all ecoregions of this ecozone. These species show a broad ecological tolerance and adaptability to the various habitats. Several species, such as *C.brevior*, *C.douglasii*, *C.duriuscula*, *C.gravida*, *C.meadii*, *C.petasata*, *C.raynoldsii*, *C.saximontana*, *C.simulata* and *C.tetanica* are present only in the Prairie ecozone, which demonstrates their narrow ecological tolerance. The Moist Mixed Grassland has the highest biodiversity index (4.04), suggesting a diversified and balanced environment with a wide range of species. The Mixed Grassland has the lowest diversity score (3.66), indicating a larger dominance of certain species and fewer species overall. The Aspen Parkland and Cypress Upland have diversity levels of 4.01 and 3.74, respectively. Overall, the Prairie ecozone has high diversity indices, indicating a well-balanced diversity amongst its ecoregions with minor variance.

The Boreal Plain ecozone displays the highest *Carex* species count, particularly in the Mid-Boreal Upland, which hosts 71 species. The Mid-Boreal Lowland has 40 species and the Boreal Transition has 70 species. A large number of species (33) are present in all three ecoregions, highlighting their adaptability to the diverse conditions. On the other hand, the group of sedges with a limited distribution within this ecozone includes 13 species. Some other species, like *C.cristatella*, *C.leptonervia*, *C.mackenziei*, *C.pedunculata*, *C.projecta* and *C.sterilis*, are unique to the Boreal Plain ecozone. The Boreal Transition and Mid-Boreal Upland have the highest diversity indices of 4.26 and 4.25, respectively. These values suggest that both ecoregions hold a rich and balanced species composition, contributing to a high ecosystem stability and resilience. The Mid-Boreal Lowland, with a diversity index of 3.69, shows lower diversity compared to the other two ecoregions.

The Boreal Shield ecozone shows a moderate *Carex* species richness, with the Athabasca Plain possessing 36 species and Churchill River Upland having 28 species. Multiple species (14) are commonly found in both the Athabasca Plain and Churchill River Upland. At the same time, *C.heleonastes* and *C.maritima* are present only in this ecozone. The Athabasca Plain ecoregion has a slightly higher diversity index (3.58) compared to the Churchill River Upland, which has a diversity index of 3.45. This indicates that the Athabasca Plain has a more balanced species composition.

The Taiga Shield ecozone generally exhibits a lower *Carex* species count compared to others, with both the Selwyn Lake Upland and Tazin Lake Upland hosting 30 species. Several species (21) are prevalent in both ecoregions, demonstrating their ability to thrive under the harsher conditions of this ecozone. Such species as *C.arctogena*, *C.bicolor*, *C.bigelowii*, *C.glacialis* and C.supinasubsp.spaniocarpa are found only in the Taiga Shield ecozone, known from one or both ecoregions.

There are 11 *Carex* species with a wide distribution in all four ecozones and at least in nine out of eleven ecoregions in the Province (Suppl. material 2). These are *C.aquatilis*, *C.aurea*, *C.capillaris*, *C.diandra*, *C.disperma*, *C.foenea*, C.inopssubsp.heliophila, *C.leptalea*, *C.rostrata*, *C.siccata* and *C.utriculata*, indicating their high ecological flexibility. Two of these species (*C.aquatilis* and *C.siccata*) are widespread across all 11 ecoregions.

There are also 12 *Carex* species that have a restricted distribution, known only in one ecozone (Suppl. material 2). This group includes *C.arcta*, *C.arctogena*, *C.bicolor*, *C.bigelowii*, *C.cristatella*, *C.heleonastes*, *C.leptonervia*, *C.mackenziei*, *C.maritima*, *C.petasata*, *C.raynoldsii* and *C.tetanica*. These *Carex* species should be considered for conservation along with other sedges with low occurrence records in the Province.

## Discussion

The genus *Carex* is most diverse in the Northern Hemisphere boreo‐temperate zone and, to a lesser extent, in the Southern Hemisphere temperate zone (Martin-Bravo et al. 2019). In North America, sedges are commonly associated with moist to wet habitats, usually with water not more than 50 cm deep in the growing season (Ball and Reznicek 2002). Considering the diversity of the genus and its ecological importance, *Carex* provides a good opportunity to understand the role of geography and ecology in the plant speciation (Spalink et al. 2016).

This study provides a comprehensive overview of the diversity, distribution and habitat preferences of *Carex* species relative to environmental conditions in Saskatchewan. This research supplies the empirical evidence needed to support the previously suggested theory of the biogeographical differentiation of the genus *Carex* in the Province (Danylyk and Kricsfalusy 2020). Moreover, the results of the current analysis of the *Carex* specimen-based occurrences demonstrate that most sedges are geographically and ecologically widespread in Saskatchewan, with high diversity unevenly distributed across and within all four ecozones or biomes. The central part of the Province possesses the highest number of *Carex* species, which corresponds to the middle elevation range and the medium temperature range. The conducted analysis indicates that *Carex* species distribution is consistent across different precipitation ranges, but their highest number is observed in the high precipitation range in the central part of Saskatchewan. Regarding the climate moisture index, which illustrates the relationship between plant water demand and available precipitation, *Carex* species are distributed under wet conditions and they also have significant representation under moist conditions. In relation to soil properties, our findings suggest that *Carex* species commonly occur in the Chernozemic and Luvisolic orders, with notable representation in the Brunisolic and Regosolic orders as well.

The total evaluation suggests that the central part of Saskatchewan shows the highest number of *Carex* species, corresponding mostly with the ecoregions within the Boreal Plain and Prairie ecozones and partially within the Boreal Shield ecozone. The area of the highest species richness encompasses the Mid-Boreal Upland and Boreal Transition ecoregions within the Boreal Plain ecozone. It is likely that the areas located in the most southern and northern parts of the Province apply ecological ﬁlters on the *Carex* species established within them. Our research reveals that the diversification and species richness of sedges in Saskatchewan are associated with temperature and precipitation and, to a lesser extent, soil evolution. Similar patterns of sedge diversity were documented in other regions of North America (Voss and Reznicek 2012, Kartesz 2013, Spalink et al. 2016). Our study also complements recent biogeographical works on the spatial diversity of Cyperaceae in North America (Spalink et al. 2016, Pender et al. 2021).

Spatial patterns of *Carex* distribution in Saskatchewan tend to exhibit proximity to major cities and towns in the Province, as well as to locations of main roads and highways, suggesting the potential of collection biases. Additional challenges that could have had an impact on *Carex* specimen collection in northern Saskatchewan might include small number of individuals participating in fieldwork and low density of collections across a large geographical region. The excessive cost of travel to the northern Saskatchewan might have also caused collecting to be severely restricted. A bias against the collection of small plants and the difficulty to identify species should be noted as well. Together, all these factors may have had influenced collection practices and lead to biases in *Carex* specimen collection in ways that affect our analysis.

The under-sampled regions of Saskatchewan should be prioritised for future surveys of *Carex* species. The result of the current sedge distribution mapping could serve as an important input in the regional assessments of threatened species recommended by IUCN (2012) since they help determine some of the parameters for the evaluation by allowing for the calculation of Area of Occupancy (AOO) and Extent of Occurrence (EOO). Overall, despite knowledge gaps and sampling biases, the data obtained in this study allowed the identification of centres of sedge richness and, on the other hand, species with a restricted distribution in Saskatchewan that should be considered priority targets for the genus *Carex* conservation.

## Conclusions

The results of the current ecological study using geospatial analysis allow us to distinguish the central part of Saskatchewan as a heterogeneous region with optimal conditions for *Carex* species, including a sufficient amount of moisture and favourable temperatures. These ecological parameters characterise the Boreal Plain and partially Prairie ecozones, which are inextricably linked with the geographic ranges of *Carex* species in Saskatchewan. Establishing the nature of the distribution of sedges in the Province from general range regularities to regional ecological features supports the validity of the chosen methodology, which makes it possible to independently assess the distribution of species using different methods (biogeographical and ecological). While delineating the main environmental factors driving the distribution of sedges, we must also consider the complex nature of a large number of other diverse factors, in particular, of a biogeographical nature.

Our integrative approach proved to be effective in describing *Carex* species diversity, distribution patterns and habitat preferences in Saskatchewan. This approach enabled a data-driven study where spatial and non-spatial analyses and visualisations provided additional working tools for biodiversity assessment. The combined analysis of data obtained from various sources minimised the biases associated with each of the datasets and helped to improve the quality of this assessment. Combining different biodiversity dimensions revealed new distribution patterns disclosing the relative roles of historical and environmental factors in shaping *Carex* diversity in Saskatchewan. Understanding these distribution patterns and ecological requirements of sedges are essential for assessing conservation status, habitat modelling studies, providing information for conservation strategies and enhancing biodiversity resilience in the face of environmental changes.

## Supplementary Material

FFDC7565-B48F-5C6D-A5B3-FE4730C471B2 10.3897/BDJ.13.e144840.suppl1 Supplementary material 1 Summary of the geospational analysis of the Carex specimen-based distribution in Saskatchewan. Data type Spatial. Brief description Statistics data including the *Carex* specimen-based occurrences, species richness, 50 km x 50 km UTM grids with species, 50 km x 50 km UTM grids with hotspots and species in hotspots. File: oo_1267955.docx https://binary.pensoft.net/file/1267955 Vladimir Kricsfalusy, Austin Godfrey, Kakon Chakma, Andrew Stewart, Ivan Danylyk.

15EA924B-18C1-5185-8F27-C2399F990A72 10.3897/BDJ.13.e144840.suppl2 Supplementary material 2 Distribution of the Carex specimen-based occurrences in different ecosystems of Saskatchewan. Data type Occurrence distribution. Brief description Distribution of the *Carex* specimen-based occurrences in different ecosystems of Saskatchewan, including four ecozones or biomes and 11 ecoregions. File: oo_1267954.docx https://binary.pensoft.net/file/1267954 Vladimir Kricsfalusy, Austin Godfrey, Kakon Chakma, Andrew Stewart, Ivan Danylyk.

## Figures and Tables

**Table 1.** Selected environmental variables that determine *Carex* species distribution in Saskatchewan.

**S/N**	**Code**	**Environmental Variable**
1	ELEV	Elevation (m)
2	MAT	Mean Annual Temperature (°C)
3	MAP	Mean Annual Precipitation (mm)
4	CMI	Climate Moisture Index
5	SOIL	Soil Order: Brunisolic, Chernozemic, Gleysolic, Luvisolic, Organic, Regosolic, Solonetzic, Vertisolic, and Unidentified
6	ECOZ	Ecozone: Prairie, Boreal Plain, Boreal Shield, and Taiga Shield
7	ECOR	Ecoregion: Aspen Parkland, Athabasca Plain, Boreal Transition, Churchill River Upland, Cypress Upland, Mid-Boreal Lowland, Mid-Boreal Upland, Mixed Grassland, Moist Mixed Grassland, Selwyn Lake Upland, and Tazin Lake Upland
