# Supplementary material for: Evaluating the diversity, distribution patterns and habitat preferences of Carex species (Cyperaceae) in western Canada using geospatial analysis
Source: Biodivers Data J. 2025 Apr 30;13:e144840. doi: 10.3897/BDJ.13.e144840 (PMC12059577; doi:10.3897/BDJ.13.e144840)
Supplement: Supplementary material 1 — Summary of the geospational analysis of the Carex specimen-based distribution in Saskatchewan. [file bdj-13-e144840-s001.docx]

| Environmental variable | Environmental variable ranges | Species occurrences (Mean ± SD) | | Total species  occurrences | | Species richness | | Grids with species | | Grids with hotspots | | Species in hotspots | |  |
| --- | --- | --- | --- | --- | --- | --- | --- | --- | --- | --- | --- | --- | --- | --- |
| Elevation (ELEV), m | 206-455 | | 11.43±14.23 | | 994 | | 87 | | 68 | | 3 | | 3 | |
|  | 456-640 | | 13.44±11.26 | | 1250 | | 93 | | 91 | | 8 | | 5 | |
|  | 641-1386 | | 6.04±5.83 | | 411 | | 68 | | 50 | | 0 | | 0 | |
| Mean Annual Temperature (MAT), *^0^*C | <= 1 | | 9.40±9.46 | | 494 | | 54 | | 40 | | 0 | | 0 | |
|  | 2-10 | | 13.33±13.74 | | 1176 | | 88 | | 269 | | 6 | | 3 | |
|  | 11-46 | | 13.86±13.15 | | 985 | | 71 | | 88 | | 4 | | 1 | |
| Mean Annual Precipitation (MAP), mm | 282-396 | | 9.38±10.26 | | 962 | | 89 | | 81 | | 3 | | 1 | |
|  | 397-461 | | 9.85±10.21 | | 1014 | | 93 | | 81 | | 4 | | 3 | |
|  | 462-547 | | 6.54±8.99 | | 673 | | 71 | | 166 | | 3 | | 2 | |
| Climate Moisture Index (CMI) | 2-3 | | 9.48±9.00 | | 617 | | 65 | | 37 | | 3 | | 1 | |
|  | 4-5 | | 9.79±7.40 | | 766 | | 80 | | 40 | | 2 | | 2 | |
|  | 6-7 | | 11.04±11.36 | | 897 | | 81 | | 46 | | 2 | | 1 | |
|  | 8-9 | | 5.75±5.84 | | 374 | | 63 | | 34 | | 2 | | 1 | |
| Soil Order (SOIL) | Brunisolic | | 6.26±7.66 | | 426 | | 68 | | 77 | | 1 | | 1 | |
|  | Chernozemic | | 11.81±11.38 | | 921 | | 78 | | 13 | | 3 | | 2 | |
|  | Gleysolic | | 1.00±0.00 | | 7 | | 7 | | 6 | | 0 | | 0 | |
|  | Luvisolic | | 7.24±5.97 | | 543 | | 75 | | 48 | | 4 | | 2 | |
|  | Organic | | 2.52±2.16 | | 136 | | 54 | | 43 | | 1 | | 1 | |
|  | Regosolic | | 3.45±2.32 | | 238 | | 69 | | 54 | | 1 | | 2 | |
|  | Solonetzic | | 1.14±0.35 | | 16 | | 14 | | 10 | | 0 | | 0 | |
|  | Vertisolic | | 2.09±0.97 | | 48 | | 23 | | 28 | | 0 | | 0 | |
| Ecozone (ECOZ) | Prairie | | 11.53±11.12 | | 964 | | 68 | | 88 | | 2 | | 2 | |
|  | Boreal Plain | | 14.18±13.43 | | 980 | | 85 | | 61 | | 4 | | 3 | |
|  | Boreal Shield | | 9.33±10.63 | | 48 | | 61 | | 47 | | 1 | | 1 | |
|  | Taiga Shield | | 3.64±2.29 | | 964 | | 39 | | 29 | | 0 | | 0 | |
| Ecoregion (ECOR) | Aspen Parkland | | 5.38±4.47 | | 295 | | 55 | | 31 | | 0 | | 0 | |
|  | Moist Mixed Grassland | | 5.33±5.33 | | 358 | | 57 | | 24 | | 2 | | 1 | |
|  | Mixed Grassland | | 7.64±7.40 | | 207 | | 39 | | 33 | | 0 | | 0 | |
|  | Cypress Upland | | 2.00±0.97 | | 102 | | 42 | | 40 | | 0 | | 0 | |
|  | Mid-Boreal Lowland | | 2.45±1.45 | | 67 | | 40 | | 34 | | 0 | | 0 | |
|  | Mid-Boreal Upland | | 1.68±0.93 | | 534 | | 70 | | 41 | | 2 | | 1 | |
|  | Boreal Transition | | 6.46±7.63 | | 372 | | 71 | | 20 | | 3 | | 3 | |
|  | Athabasca Plain | | 4.72±4.19 | | 169 | | 36 | | 21 | | 0 | | 0 | |
|  | Churchill River Upland | | 6.30±4.75 | | 400 | | 28 | | 45 | | 1 | | 1 | |
|  | Selwyn Lake Upland | | 2.77±1.36 | | 60 | | 31 | | 30 | | 0 | | 0 | |
|  | Tazin Lake Upland | | 5.25±3.56 | | 82 | | 30 | | 21 | | 0 | | 0 | |
