# Supplementary material for: Evaluating the diversity, distribution patterns and habitat preferences of Carex species (Cyperaceae) in western Canada using geospatial analysis
Source: Biodivers Data J. 2025 Apr 30;13:e144840. doi: 10.3897/BDJ.13.e144840 (PMC12059577; doi:10.3897/BDJ.13.e144840)
Supplement: Supplementary material 2 — Distribution of the Carex specimen-based occurrences in different ecosystems of Saskatchewan. [file bdj-13-e144840-s002.docx]

| **Ecozone:** Prairie, Boreal Plain, Boreal Shield, Taiga Shield.  **Ecoregion:** AP – Aspen Parkland, ATP – Athabasca Plain, BT – Boreal Transition, CRU – Churchill River Upland, CU – Cypress Upland, MBL – Mid-Boreal Lowland, MBU – Mid-Boreal Upland, MG – Mixed Grassland, MMG – Moist Mixed Grassland, SLU – Selwyn Lake Upland, TLU – Tazin Lake Upland.  Fr – species frequency, *r* – species richness, *H* – Shannon–Wiener diversity index. | | | | | | | | | | | | | | |
| --- | --- | --- | --- | --- | --- | --- | --- | --- | --- | --- | --- | --- | --- | --- |
| Scientific Name | Ecosystem | | | | | | | | | | | Ecozone, *Fr* | Ecoregion, *Fr* | |
|  | Prairie | | | | Boreal Plain | | | Boreal Shield | | Taiga Shield | |  |  |  |
|  | AP | MMG | MG | CU | MBL | MBU | BT | ATP | CRU | SLU | TLU |  | |  |
| *C. adusta* |  |  | + |  |  | + | + |  |  |  |  | 2 | 3 | |
| *C. alopecoidea* | + | + | + |  | + |  | + |  |  |  |  | 2 | 5 | |
| *C. aquatilis* | + | + | + | + | + | + | + | + | + | + | + | 4 | 11 | |
| *C. arcta* |  |  |  |  |  | + |  |  |  |  |  | 1 | 1 | |
| *C. arctogena* |  |  |  |  |  |  |  |  |  | + |  | 1 | 1 | |
| *C. assiniboinensis* | + | + |  |  | + |  |  |  |  |  |  | 2 | 3 | |
| *C. atherodes* | + | + | + | + | + | + | + |  | + |  |  | 3 | 8 | |
| *C. athrostachya* | + | + | + | + |  |  | + |  |  |  |  | 2 | 5 | |
| *C. atratiformis* |  |  |  |  |  | + | + |  |  |  |  | 1 | 2 | |
| *C. aurea* | + | + | + | + | + | + | + |  | + |  | + | 4 | 9 | |
| *C. backii* | + | + | + | + | + | + | + |  |  |  |  | 2 | 7 | |
| *C. bebbii* | + | + |  | + | + | + | + |  | + |  |  | 3 | 7 | |
| *C. bicolor* |  |  |  |  |  |  |  |  |  | + |  | 1 | 1 | |
| *C. bigelowii* |  |  |  |  |  |  |  |  |  | + |  | 1 | 1 | |
| *C. brevior* | + | + | + | + |  |  |  |  |  |  |  | 1 | 4 | |
| *C. brunnescens* |  |  |  |  | + | + | + | + |  | + | + | 3 | 6 | |
| *C. buxbaumii* | + | + |  |  | + |  |  | + | + |  | + | 4 | 6 | |
| *C. canescens* |  |  |  | + | + | + | + | + |  | + | + | 4 | 7 | |
| *C. capillaris* | + | + |  | + |  | + | + | + | + | + | + | 4 | 9 | |
| *C. capitata* |  |  |  |  | + | + |  |  |  |  |  | 1 | 2 | |
| *C. chordorrhiza* | |  |  |  | + | + | + | + |  | + | + | 3 | 6 | |
| *C. cocinna* | + |  |  | + | + | + | + |  |  |  | + | 3 | 6 | |
| *C. crawei* | + | + | + |  |  |  | + |  |  |  |  | 2 | 4 | |
| *C. crawfordii* |  |  |  | + | + | + | + |  |  |  |  | 2 | 4 | |
| *C. cristatella* |  |  |  |  |  | + |  |  |  |  |  | 1 | 1 | |
| *C. cryptolepis* |  |  |  |  | + | + |  | + |  |  |  | 2 | 3 | |
| *C. deflexa* |  |  |  |  | + | + | + | + |  | + | + | 3 | 6 | |
| *C. deweyana* | + | + | + | + | + | + | + |  | + |  |  | 3 | 8 | |
| *C. diandra* | + | + |  | + | + | + | + | + | + | + | + | 4 | 10 | |
| *C. disperma* | + | + | + | + | + | + | + | + | + |  | + | 4 | 10 | |
| *C. douglasii* | + | + | + | + |  |  |  |  |  |  |  | 1 | 4 | |
| *C. duriuscula* | + | + | + | + |  |  |  |  |  |  |  | 1 | 4 | |
| *C. eburnea* | + | + | + |  |  |  | + | + |  |  | + | 4 | 6 | |
| *C. echinata* |  |  |  |  |  |  | + | + |  |  |  | 2 | 2 | |
| *C. filifolia* | + | + | + | + |  |  | + |  |  |  |  | 2 | 5 | |
| *C. foenea* | + | + | + | + |  | + | + | + | + | + | + | 4 | 10 | |
| *C. garberi* | + | + |  | + |  | + | + | + | + | + |  | 4 | 8 | |
| *C. glacialis* |  |  |  |  |  |  |  |  |  | + | + | 1 | 2 | |
| *C. granularis* | + |  |  |  |  |  | + |  |  |  |  | 2 | 2 | |
| *C. gravida* |  | + | + |  |  |  |  |  |  |  |  | 1 | 2 | |
| *C. heleonastes* |  |  |  |  |  |  |  |  | + |  |  | 1 | 1 | |
| *C. hoodii* |  |  |  | + |  | + |  |  |  |  |  | 2 | 2 | |
| *C. hookerana* |  |  | + | + |  |  | + |  |  |  |  | 2 | 3 | |
| *C. houghtoniana* | |  |  |  |  | + | + |  |  |  |  | 1 | 2 | |
| *C. hystrincina* | + | + | + |  |  | + | + |  |  |  |  | 2 | 5 | |
| *C. inops* | + | + | + | + | + | + | + |  | + | + |  | 4 | 9 | |
| *C. interior* | + | + |  | + | + | + | + | + | + |  |  | 3 | 8 | |
| *C. lacustris* |  |  |  |  | + | + | + |  |  |  |  | 1 | 3 | |
| *C. laeviconica* | + | + | + |  |  |  | + |  |  |  |  | 2 | 4 | |
| *C. lasiocarpa* |  | + |  |  | + | + | + | + |  |  |  | 3 | 5 | |
| *C. lenticularis* |  |  |  |  |  |  |  | + |  |  | + | 2 | 2 | |
| *C. leptalea* | + | + |  | + | + | + | + | + | + | + |  | 4 | 9 | |
| *C. leptonervia* |  |  |  |  |  | + |  |  |  |  |  | 1 | 1 | |
| *C. limosa* |  | + |  |  |  | + | + | + |  | + |  | 4 | 5 | |
| *C. livida* |  | + |  |  |  | + | + | + |  | + |  | 4 | 5 | |
| *C. loliacea* |  |  |  |  |  | + |  |  |  | + | + | 2 | 3 | |
| *C. mackenziei* |  |  |  |  |  | + |  |  |  |  |  | 1 | 1 | |
| *C. magellanica* |  |  |  |  | + | + | + | + |  | + | + | 3 | 6 | |
| *C. maritima* |  |  |  |  |  |  |  | + |  |  |  | 1 | 1 | |
| *C. meadii* | + | + |  |  |  |  |  |  |  |  |  | 1 | 2 | |
| *C. media* |  |  |  |  | + | + | + |  |  | + | + | 2 | 5 | |
| *C. michauxiana* | |  |  |  |  |  |  | + |  | + |  | 2 | 2 | |
| *C. microptera* | + | + | + | + |  | + | + |  |  |  |  | 2 | 6 | |
| *C. nardina* |  |  |  |  |  | + | + |  |  | + | + | 2 | 4 | |
| *C. obtusata* | + | + |  | + |  | + | + |  |  |  |  | 2 | 5 | |
| *C. oederi* | + | + | + | + | + | + | + |  |  |  | + | 3 | 8 | |
| *C. oligosperma* |  |  |  |  |  | + |  | + |  |  |  | 2 | 2 | |
| *C. parryana* | + | + | + |  |  | + | + |  | + |  |  | 3 | 6 | |
| *C. pauciflora* |  |  |  |  |  | + |  | + |  |  |  | 2 | 2 | |
| *C. peckii* | + | + |  |  | + | + | + |  | + |  |  | 3 | 6 | |
| *C. pedunculata* |  |  |  |  | + | + |  |  |  |  |  | 1 | 2 | |
| *C. pellita* | + | + | + | + | + | + | + |  |  |  |  | 2 | 7 | |
| *C. petasata* |  |  |  | + |  |  |  |  |  |  |  | 1 | 1 | |
| *C. praegracilis* | + | + | + |  |  | + | + |  |  |  |  | 2 | 5 | |
| *C. prairea* | + | + |  |  |  | + | + |  |  |  |  | 2 | 4 | |
| *C. praticola* | + | + | + | + |  | + | + |  | + |  | + | 4 | 8 | |
| *C. projecta* |  |  |  |  |  | + | + |  |  |  |  | 1 | 2 | |
| *C. pseudo-cyperus* | |  |  |  | + | + | + |  |  |  |  | 1 | 3 | |
| *C. raynoldsii* |  |  |  | + |  |  |  |  |  |  |  | 1 | 1 | |
| *C. retrorsa* | + | + |  |  | + | + | + |  | + |  |  | 3 | 6 | |
| *C. richardsonii* | + |  |  |  |  | + | + | + | + |  |  | 3 | 5 | |
| *C. rossii* | + | + | + | + |  | + | + | + | + |  |  | 3 | 8 | |
| *C. rostrata* |  | + | + | + | + | + | + | + |  | + | + | 4 | 9 | |
| *C. sartwellii* | + | + |  |  |  | + | + |  |  |  |  | 2 | 4 | |
| *C. saxatilis* |  |  |  |  |  |  |  | + |  | + | + | 2 | 3 | |
| *C. saximontana* | + | + | + | + |  |  |  |  |  |  |  | 1 | 4 | |
| *C. scirpoidea* | + | + | + |  | + |  | + | + |  | + | + | 4 | 8 | |
| *C. siccata* | + | + | + | + | + | + | + | + | + | + | + | 4 | 11 | |
| *C. simulata* | + | + | + | + |  |  |  |  |  |  |  | 1 | 4 | |
| *C. sprengelii* | + | + | + | + |  |  | + |  |  |  |  | 2 | 5 | |
| *C. sterilis* |  |  |  |  |  | + | + |  |  |  |  | 1 | 2 | |
| *C. stipata* | + | + |  | + | + | + | + |  | + |  |  | 3 | 7 | |
| *C. supina* |  |  |  |  |  |  |  |  |  | + | + | 1 | 2 | |
| *C. synchnocephala* | + | + | + |  |  | + | + |  | + |  |  | 3 | 6 | |
| *C. tenera* | + | + | + | + |  | + | + |  | + |  |  | 3 | 7 | |
| *C. tenuiflora* |  |  |  |  | + | + | + | + |  |  | + | 3 | 5 | |
| *C. tetanica* |  | + |  |  |  |  |  |  |  |  |  | 1 | 1 | |
| *C. tonsa* |  |  | + |  | + | + | + | + |  |  |  | 3 | 5 | |
| *C. torreyi* | + | + | + | + |  |  | + |  |  |  |  | 2 | 5 | |
| *C. trisperma* |  |  |  |  | + | + | + | + |  |  |  | 2 | 4 | |
| *C. umbellata* |  |  |  |  |  | + |  | + |  | + | + | 3 | 4 | |
| *C. utriculata* | + | + |  | + | + | + | + | + | + | + | + | 4 | 10 | |
| *C. vaginata* | + | + |  |  |  | + | + |  | + | + | + | 4 | 7 | |
| *C. vulpinoidea* | + | + |  |  |  | + | + |  | + |  |  | 3 | 5 | |
| *C. xerantica* | + | + | + | + |  |  | + |  | + |  |  | 3 | 6 | |
| Species richness (*r*) | 55 | 57 | 39 | 42 | 40 | 70 | 71 | 36 | 29 | 30 | 30 |  |  | |
| Shannon–Wiener diversity index (*H*) | 4.01 | 4.04 | 3.66 | 3.74 | 3.69 | 4.25 | 4.26 | 3.58 | 3.45 | 3.40 | 3.40 |  |  | |
|  | | | | | | | | | | | | | | |
